# Antibiotic Exposure Does Not Impact Anti-BRAF/Anti-MEK Targeted Therapy Outcome in Patients with Advanced Melanoma

**DOI:** 10.3390/curroncol32110630

**Published:** 2025-11-10

**Authors:** Yu Shi Wang, Qing Yin Wang, Alexia Erika Moise, Hamida Claudia Syed, Julie Malo, Spencer Soberano, Wiam Belkaid, Meriem Messaoudene, Karl Bélanger, Antoine Desilets, Rahima Jamal, Bertrand Routy, Arielle Elkrief

**Affiliations:** 1 Internal Medicine Division, Department of Medicine, Centre Hospitalier de l’Université de Montréal (CHUM), Montreal, QC H2X 1R6, Canada; yu.shi.wang@umontreal.ca (Y.S.W.); qing.yin.wang@umontreal.ca (Q.Y.W.); 2 Axe Cancer, Centre de Recherche du Centre Hospitalier de l’Université de Montréal (CRCHUM), Montreal, QC H2X 0A9, Canada; alexia.erika.moise@umontreal.ca (A.E.M.); hamida-claudia.syed.chum@ssss.gouv.qc.ca (H.C.S.); julie.malo.chum@ssss.gouv.qc.ca (J.M.); wiam.belkaid.chum@ssss.gouv.qc.ca (W.B.); meriem.messaoudene@umontreal.ca (M.M.); antoine.desilets@umontreal.ca (A.D.); bertrand.routy@umontreal.ca (B.R.); 3 Hematology-Oncology Division, Department of Medicine, Centre Hospitalier de l’Université de Montréal (CHUM), Montreal, QC H2X 1R6, Canada; karl.belanger.med@ssss.gouv.qc.ca (K.B.); rahima.jamal.med@ssss.gouv.qc.ca (R.J.)

**Keywords:** melanoma, antibiotics, gut microbiome, BRAF inhibitors, MEK inhibitors, immunotherapy

## Abstract

The gut microbiome is an established predictor of response to immune checkpoint inhibitors (ICI) in melanoma, and antibiotic exposure prior to ICI initiation is a validated negative prognostic factor. About half of melanoma patients harbor BRAF mutations and are treated with BRAF/MEK inhibitors (BRAFi/MEKi). While the detrimental impact of antibiotics is well described in the ICI setting, their effect on BRAFi/MEKi efficacy remains unknown. We retrospectively analyzed 49 advanced BRAF-mutant melanoma patients treated with BRAFi/MEKi. Antibiotic-exposed patients were compared with non-exposed patients across three time windows: within 30, 60, or 90 days before and after therapy initiation. Outcomes included progression-free survival (PFS), overall survival (OS), and overall response rate (ORR). Among the cohort, 41% had antibiotic exposure within ±30 days, 53% within ±60 days, and 57% within ±90 days. Baseline characteristics were comparable between groups, except for worse ECOG scores in antibiotic-exposed patients. Across all windows, ORR, PFS, and OS were comparable between groups. Unlike what was observed in the ICI setting, antibiotic use did not negatively affect outcomes with BRAFi/MEKi. Despite small sample size, these findings suggest that the detrimental prognostic impact of antibiotics is specific to immunotherapy, highlighting the importance of evaluating the microbiome as a predictive biomarker across treatment contexts.

## Simple Summary

Although immunotherapy has greatly improved outcomes in advanced melanoma, antibiotics can negatively alter the gut microbiome composition, thereby reducing the effectiveness of immunotherapy. In parallel, BRAF/MEK inhibitors are another key therapeutic option for BRAF-mutated melanoma. However, the impact of antibiotics on outcomes in BRAF/MEK targeted therapy is unknown. In our cohort of 49 melanoma patients treated with BRAF/MEK inhibitors, antibiotic use did not worsen outcomes (objective response rate, progression-free survival, and overall survival). Our findings suggest that antibiotics are uniquely deleterious in immunogenic contexts such as immunotherapy, as opposed to a general effect related to patient fragility. We are currently working to confirm these results in larger, independent patient groups.

## 1. Introduction

Approximately 40–50% of cutaneous melanomas harbor activating mutations in the BRAF oncogene, most commonly at codon 600, resulting in constitutive activation of the MAPK signaling pathway and promoting tumor proliferation [1,2]. The V600E substitution, in which valine is replaced by glutamic acid, accounts for 80–90% of all BRAF mutations, whereas V600K, a valine-to-lysine substitution, occurs in 5–20% of cases and is more frequently observed in older patients with chronically sun-damaged skin. Rarer variants such as V600D, V600R, and V600M have also been reported, although they remain less well characterized [3].

The development of selective BRAF V600 inhibitors (BRAFi), which specifically target the mutated BRAF protein, and their subsequent combination with MEK inhibitors (MEKi) has substantially improved outcomes in advanced melanoma. These regimens have produced durable increases in objective response rates (ORR) and significant improvements in progression-free survival (PFS) and overall survival (OS) compared to chemotherapy alone [4,5]. Pivotal trials with dabrafenib plus trametinib (COMBI-d and COMBI-v) and with encorafenib plus binimetinib (COLUMBUS) established BRAFi/MEKi combinations as standards-of-care, with durable outcomes extending to five years in pooled analyses [6,7,8,9,10]. Nonetheless, both intrinsic and acquired resistance remain major clinical challenges, with the majority of patients ultimately experiencing disease progression [5].

In parallel, research in immuno-oncology has highlighted the gut microbiome as a critical determinant of treatment response within the immunotherapy landscape. Retrospective studies have consistently demonstrated that antibiotic exposure before or after initiation of immune checkpoint inhibitors (ICI) is associated with worsened outcomes across solid tumor sites (including melanoma, non-small cell lung cancer (NSCLC), renal cell carcinoma (RCC), and urothelial carcinoma), independently of other negative prognostic factors such as line of therapy, performance status, and burden of disease [11]. A recent systematic review and meta-analysis of 130 studies which included 46,232 patients treated with ICI confirmed a consistent association between antibiotic use and inferior survival [12].

This detrimental effect is thought to be mediated by antibiotic-induced dysbiosis, resulting in diminished beneficial commensal species such as *Akkermansia muciniphila* and *Ruminococcus* spp., combined with increased relative abundance of deleterious species such as *Enterocloster bolteae*. This dysbiosis is believed to induce loss of ileal checkpoint MAdCAM-1, leading to an efflux of immunosuppressive regulatory T cells from the gut into the tumoral microenvironment, ultimately culminating in impaired antitumor immunity and ICI resistance.

Despite the robust clinical and mechanistic findings pointing to the causative role of antibiotics on ICI resistance, concerns have been raised that indication and severity biases may confound these associations in human cohort studies. As a result, it is essential to evaluate the impact of antibiotics in non-ICI contexts such as chemotherapy and targeted therapy. Currently, data is limited on the impact of antibiotics in patients receiving BRAFi/MEKi.

As patients with advanced melanoma frequently require antibiotics for infection-related complications, clarifying the potential prognostic impact of antibiotic exposure in this population has important clinical implications. Therefore, the objective of this study is to evaluate the association between antibiotic exposure and clinical outcomes in patients with advanced BRAF-mutant melanoma treated with BRAFi/MEKi.

## 2. Materials and Methods

### 2.1. Study Population

Patients were retrospectively identified from the biobank and tumor registry of the Centre hospitalier de l’Université de Montréal (CHUM) between January 2013 and December 2024. Eligible patients had pathologically confirmed BRAF-mutated advanced melanoma (stage IV or unresectable stage III) and had received first or later line BRAFi/MEKi therapy. BRAF mutation status was determined by tumor molecular testing with either next-generation sequencing (AmpliSeq 52-gene panel, Thermo Fisher Scientific, Waltham, MA, USA) or PCR, and/or immunohistochemistry; status was recorded as V600E, V600K, or other less common variants. Patients were excluded if clinical data were unavailable due to follow-up occurring outside the institution.

### 2.2. Antibiotic Exposure Assessment

Antibiotic (ATB) exposure was defined as systemic administration by oral, intravenous, or intramuscular routes. To evaluate the potential impact of exposure timing, three exposure windows were considered: within 30 days before or after BRAFi/MEKi initiation, within 60 days before or after initiation, and within 90 days before or after initiation. For each window, patients were classified as either ATB-exposed or non-exposed, and outcomes were compared between the two groups.

### 2.3. Outcomes

Outcomes of interest were PFS, OS, and ORR. PFS was defined as the time from BRAFi/MEKi initiation to radiologic or clinical disease progression or death from any cause, whichever occurred first. OS was defined as the time from BRAFi/MEKi initiation to death from any cause. For PFS and OS, patients who were progression-free or alive at the time of data cutoff were censored at the date of last follow-up. ORR was defined as the proportion of patients achieving a partial or complete response. All outcomes were investigator-assessed.

### 2.4. Statistical Analysis

Baseline demographics and clinical characteristics were compared between ATB-exposed and unexposed groups using descriptive statistics. Continuous variables were summarized as medians with interquartile ranges (IQR), and categorical variables as counts and percentages. Categorical variables were analyzed with Fisher’s exact test, and continuous variables with the Wilcoxon rank-sum test. ORR was compared between groups using Fisher’s exact test. PFS and OS were estimated with the Kaplan–Meier method and compared using the log-rank test. Hazard ratios (HRs) and 95% confidence intervals (CIs) were calculated using Cox proportional hazards models. All statistical tests were two-sided, with *p* < 0.05 considered statistically significant. Analyses were performed using R version 4.4.0 (R Foundation for Statistical Computing, Vienna, Austria).

### 2.5. Ethics

The study was conducted in accordance with institutional ethical standards and approved by the local Research Ethics Board (project number 2017-6770).

## 3. Results

### 3.1. Patient Characteristics

A total of 49 patients with BRAF-mutant advanced melanoma treated with BRAFi/MEKi were included in the analysis. Baseline characteristics are summarized in Table 1, Table 2 and Table 3 according to window of antibiotic exposure. The median age at treatment initiation was approximately 60 years, and most patients presented with stage IV disease and harbored a BRAF V600E mutation.

Notably, the Eastern Cooperative Oncology Group (ECOG) performance status was significantly worse among patients who received ATBs compared to the non-exposed group: 40% vs. 11.5% with ECOG ≥ 2 in the ±30-day cohort (*p* = 0.038) and 36% vs. 9.5% in the ±60-day cohort (*p* = 0.045). In the ±90-day cohort, the difference did not reach statistical significance (*p* = 0.092), although the proportion remained numerically higher in the ATB group (33.3% vs. 10.5%).

Other baseline features were broadly comparable between groups and across timing cohorts. The prevalence of liver metastases was similar and not statistically different. Brain metastases were numerically more frequent in the ATB group, although the difference was not statistically significant. Baseline LDH levels were slightly higher in the non-ATB group, but again without statistical significance.

Of note, among the 49 included patients, 43 (87.8%) also received ICI therapy at some point during their disease course, either as first-line treatment before BRAFi/MEKi or as a later-line therapy following targeted treatment. Only 6 patients (12.2%) never received ICI therapy.

### 3.2. Antibiotic Use

We assessed time windows of exposure to ATB at 30, 60, or 90 days prior to or after initiation of BRAFi/MEKi, as performed in previous landmark ATB exposure studies [12]. The percentage of patients exposed to ATBs according to the time windows were: 41% (20/49) within ±30 days, 53% (26/49) within ±60 days, and 57% (28/49) within ±90 days of BRAFi/MEKi initiation (Table 1, Table 2 and Table 3). The relatively high incidence of ATB exposure, compared with the 30% previously described for ICIs [12,13], likely reflects that fever is a common side effect of BRAFi/MEKi [8,9]. The most common indications for ATB use were fever without documented source, urinary tract infection, and pneumonia. Most courses were short in duration (1–7 days), though prolonged treatments were observed for deep tissue infections or latent tuberculosis (Supplemental Tables S1–S3). As previously reported, the most frequently prescribed antibiotic classes were penicillin/β-lactamase inhibitors, fluoroquinolones, and cephalosporins (Supplemental Tables S4–S6) [12,13].

### 3.3. Clinical Outcomes

**Figure 1:**
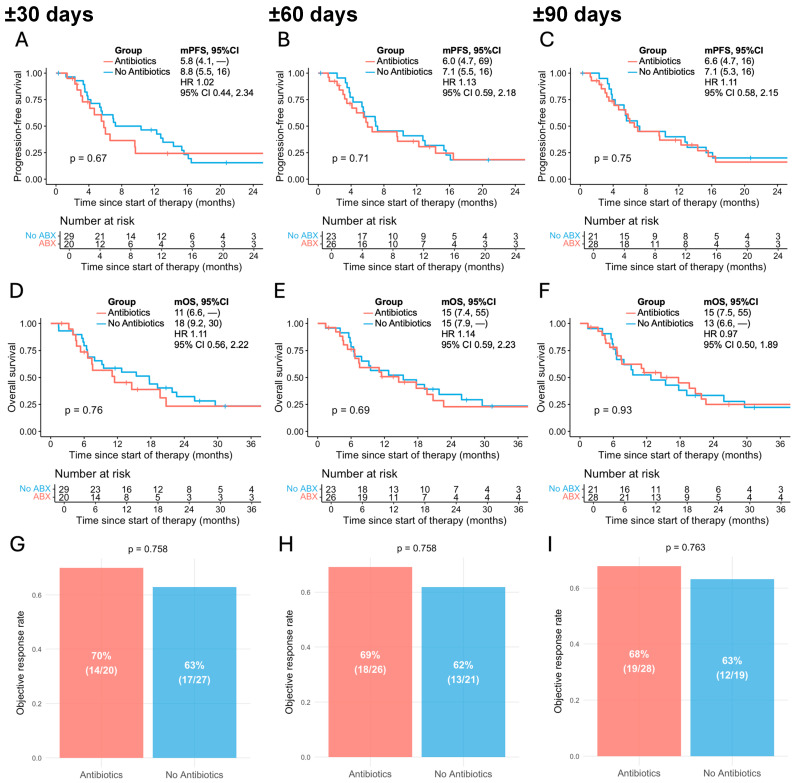
Association between antibiotic exposure and outcomes of BRAFi/MEKi in patients with advanced melanoma across different time windows relative to therapy initiation. (**A**–**C**) Progression-free survival in antibiotic-exposed vs. non-exposed patients within ±30 (**A**), ±60 (**B**), and ±90 days (**C**); (**D**–**F**) overall survival in antibiotic-exposed vs. non-exposed patients within ±30 (**D**), ±60 (**E**), and ±90 days (**F**); (**G**–**I**) objective response rate in antibiotic-exposed vs. non-exposed patients within ±30 (**G**), ±60 (**H**), and ±90 days (**I**). mPFS, median progression-free survival; mOS, median overall survival; HR, hazard ratio; 95% CI, 95% confidence interval. Median survival times are shown, with numbers in parentheses representing 95% CIs.

ATB exposure was not associated with statistically significant differences in PFS. In the ±30-day group, median PFS was 5.8 months (95% CI, 4.1–NR) in ATB-exposed patients compared with 8.8 months (95% CI, 5.5–16.0) in those unexposed (HR 1.02, 95% CI, 0.44–2.34; *p* = 0.67; Figure 1A). Similar findings were observed in the ±60-day exposed group, with median PFS of 6.0 months (95% CI, 4.7–69.0) vs. 7.1 months (95% CI, 5.5–16.0) (HR 1.13, 95% CI, 0.59–2.18; *p* = 0.71; Figure 1B). In the ±90-day analysis, median PFS was 6.6 months (95% CI, 4.7–16.0) vs. 7.1 months (95% CI, 5.3–16.0) (HR 1.11, 95% CI, 0.58–2.15; *p* = 0.75; Figure 1C).

Similarly, OS did not significantly differ between groups. In the ±30-day cohort, median OS was 11.0 months (95% CI, 6.6–NR) in ATB-exposed patients and 18.0 months (95% CI, 9.2–30.0) in unexposed patients (HR 1.11, 95% CI, 0.56–2.22; *p* = 0.76; Figure 1D). In the ±60-day cohort, median OS was 15.0 months (95% CI, 7.4–55.0) vs. 15.0 months (95% CI, 7.9–NR), respectively (HR 1.14, 95% CI, 0.59–2.23; *p* = 0.69; Figure 1E). In the ±90-day cohort, median OS was 15.0 months (95% CI, 7.5–55.0) vs. 13.0 months (95% CI, 6.6–NR), respectively (HR 0.97, 95% CI, 0.50–1.89; *p* = 0.93; Figure 1F).

The ORR did not significantly differ between ATB-exposed and unexposed groups in any of the predefined time windows. Two patients with missing ORR status were excluded from the ORR analysis. In the ±30-day cohort, ORR was 70% in the ATB-exposed group compared with 63% in the unexposed group (*p* = 0.758; Figure 1G). Similarly, in the ±60-day cohort, ORR was 69% vs. 62% (*p* = 0.758; Figure 1H), and in the ±90-day cohort, 68% vs. 63% (*p* = 0.763; Figure 1I), respectively. Across all three exposure windows, observed ORRs in both patient groups were approximately 60–70%, which is broadly comparable to the response rates reported in the COLUMBUS trial (64%) and the COMBI-v and COMBI-d trials (68%) for patients receiving BRAFi/MEKi combination therapy [8,9].

## 4. Discussion

In this retrospective cohort of patients with advanced BRAF-mutant melanoma treated with BRAFi/MEKi, systemic antibiotic exposure within 30, 60, or 90 days before and after therapy initiation was not significantly associated with inferior PFS, OS, or ORR. This study is among the first to examine the prognostic impact of antibiotics in the setting of BRAFi/MEKi therapy, and results contrast with those reported in patients receiving ICI therapy, for which antibiotic exposure has been consistently associated with inferior outcomes [11,12,13,14,15,16,17].

In the immunotherapy setting, antibiotic exposure has been repeatedly identified as an independent negative prognostic factor across multiple tumor types. In advanced melanoma, antibiotic exposure has been associated with worse outcomes in patients treated with ICI [11], findings that were validated as part of a systematic review and meta-analysis including over 46,000 patients across solid tumors, including melanoma, NSCLC, and RCC [12]. Consistent results have also been reported by multicenter analyses in NSCLC [15] and by a melanoma-specific meta-analysis pooling over 5000 patients [17], further consolidating antibiotics as a clinically relevant negative prognostic marker in the immunotherapy setting.

The proposed mechanism is antibiotic-induced dysbiosis of the gut microbiota, characterized by depletion of beneficial commensals such as *Akkermansia muciniphila* and *Ruminococcus* spp. and overgrowth of potentially pathogenic species such as *Enterocloster bolteae* [16]. This imbalance suppresses ileal expression of mucosal addressin cell adhesion molecule-1 (MAdCAM-1), which normally retains α4β7-expressing FoxP3^+^RORγt^+^ regulatory T (Treg17) cells within the intestinal lamina propria. Downregulation of MAdCAM-1 promotes trafficking of these immunosuppressive Treg17 cells from the gut to the tumor microenvironment, as demonstrated in murine models [18]. This redistribution may impair antitumor immune responses and reduce ICI efficacy, thereby providing a biologically plausible mechanism for the observed association between antibiotic exposure and poor clinical outcomes in ICI-treated patients.

Clinical data further support a causal role, as restoration of gut microbial diversity through fecal microbiota transplantation (FMT) in humans has been shown to partially reverse worsened outcomes associated with ATB exposure in melanoma patients on ICI [19,20]. Early phase trials have provided clinical evidence that FMT can restore sensitivity to ICIs in resistant melanoma or help reduce the risk of primary ICI resistance. Most recently, a phase I trial combining oral encapsulated FMT from healthy donors with first-line anti-PD-1 therapy reported an ORR of 65% and improved PFS and OS [21]. These findings reinforce the role of the gut microbiome as a modulator of ICI efficacy.

Nevertheless, concerns regarding residual confounding by indication and severity have been raised, as patients requiring antibiotics often present with baseline differences such as poorer performance status. A previous study attempted to address this concern by including a comparator cohort treated with tyrosine kinase inhibitors (TKIs) and reported that antibiotic exposure was associated with survival outcomes similar to those observed with ICIs [22]. However, the TKI group encompassed heterogeneous agents, including EGFR, ALK, and BRAF/MEK inhibitors, which act through distinct mechanisms and therefore limit interpretability. Similarly, another study suggested that antibiotic use was associated with reduced TKI efficacy in advanced melanoma and NSCLC [23], but the TKI cohort also included both EGFR and BRAF inhibitors, precluding class-specific conclusions.

By contrast, other studies did demonstrate treatment-specific differences: Cortellini et al. found that antibiotics were associated with an adverse prognostic effect in NSCLC patients treated with ICIs but not in chemotherapy cohorts of comparable size [15]; Lalani et al. reported that antibiotic use was significantly associated with worse outcomes in metastatic renal cell carcinoma patients receiving ICIs and, to a lesser extent, in those treated with interferon or VEGF-targeted therapy following cytokines, but not in those treated with mTOR inhibitors or VEGF-targeted therapy alone [24]. Collectively, these findings suggest that antibiotic-related effects are most pronounced in immune-driven therapies and are most consistently observed with ICIs, which is unlikely to be attributable to bias alone.

Whether a similar pattern exists in patients receiving targeted therapies such as BRAFi/MEKi has not previously been investigated. Unlike ICIs, these agents act primarily by inhibiting tumor cell MAPK signaling rather than directly modulating host immunity. In the present study, no detrimental association between antibiotic exposure and BRAFi/MEKi outcomes was observed. The absence of such an association in the BRAFi/MEKi setting further supports the interpretation that the impact of antibiotics on ICI survival outcomes reflects a treatment-specific interaction rather than residual bias. However, the limited sample size of the present study may have masked modest effects of antibiotics on BRAFi/MEKi outcomes. While their primary mechanism remains MAPK inhibition, preclinical and translational studies have shown that BRAFi/MEKi can remodel the tumor microenvironment by enhancing tumor antigen expression and T-cell infiltration [25,26,27]. These hypotheses warrant verification in independent cohorts with larger sample sizes and potentially also in murine models.

Several limitations of this study should be acknowledged. The relatively small cohort size limits statistical power and may have precluded detection of small differences. Another limitation is that, given the retrospective nature of our study, antibiotic exposure outside the hospital setting may not have been fully captured, leading to potential misclassification of exposure. Such non-differential misclassification typically biases results toward the null and may therefore have obscured modest associations. Moreover, microbiome sequencing was not available in this retrospective setting, preventing direct assessment of dysbiosis and its correlation with outcomes.

In addition, most patients in our cohort (88%) received immunotherapy at some point during their disease course, either as first-line treatment before BRAFi/MEKi or as later-line therapy following targeted treatment. Only 6 of the 49 patients never received ICI therapy, which limited the feasibility of a separate analysis in this subgroup. Given that antibiotic exposure is known to negatively impact outcomes in the context of ICI therapy, the high prevalence of prior or subsequent ICI exposure in our cohort may have contributed to the non-significant numerical difference observed toward worse outcomes in the antibiotic-exposed group within the ±30-day window. It cannot be excluded that antibiotic exposure may have affected the late response to immunotherapy administered prior to BRAFi/MEKi, or may have persisted and influenced the efficacy of subsequent immune-based treatments. These considerations underscore the potential confounding introduced by sequential exposure to immune-modulating therapies.

Overall, our findings provide preliminary reassurance that antibiotic exposure does not significantly compromise the efficacy of BRAFi/MEKi, an important consideration since infections are common in this patient population and appropriate antibiotic use remains essential. In addition, our study reinforces that antibiotic-induced resistance appears to be specific to ICIs and other immune-system based therapies, such as bone marrow transplantation (BMT) [28,29] and chimeric antigen receptor T-cell therapy (CAR-T) [30,31,32], rather than simply reflecting severity bias. However, previously reported immunomodulatory effects of MAPK inhibition suggest that interactions between antibiotics, the microbiome, and targeted therapy merit further study. Larger cohorts will be needed to confirm these observations and to clarify whether specific antibiotic classes, prolonged courses, or repeated exposures exert measurable influence on targeted therapy outcomes.

## 5. Conclusions

In our cohort, antibiotic exposure was not associated with inferior outcomes in advanced melanoma treated with BRAFi/MEKi. This finding adds to the growing evidence that antibiotic-mediated dysbiosis is a treatment-specific negative prognostic factor in the context of ICI therapy rather than a universal outcome due to bias. Our results also provide reassurance for judicious use of ATB in patients treated with BRAFi/MEKi, in whom infections are frequent. Ongoing validation in independent cohorts will be important to confirm these observations.

## Figures and Tables

**Table 1:** Baseline Characteristics According to Antibiotic Exposure ±30 days.

Characteristic	ABX Within 30 Days *n* = 20 ^1^	No ABX Within 30 Days *n* = 29 ^1^	*p*-Value ^2^
**Age**	62.7 [51.1, 67.0]	57.3 [44.1, 63.8]	0.5
**Sex**			>0.9
Female	7 (35.0%)	10 (34.5%)	
Male	13 (65.0%)	19 (65.5%)	
**Stage**			0.7
2 or 3 (unresectable)	2 (10.0%)	5 (17.2%)	
4 (advanced)	18 (90.0%)	24 (82.8%)	
**BRAF status**			>0.9
V600E	19 (95.0%)	26 (92.9%)	
V600K	1 (5.0%)	2 (7.1%)	
Missing	0	1	
**ECOG**			0.038
≥2	8 (40.0%)	3 (11.5%)	
0–1	12 (60.0%)	23 (88.5%)	
Missing	0	3	
**Melanoma type**			0.5
Cutaneous	16 (80.0%)	26 (89.7%)	
Mucosal	1 (5.0%)	0 (0.0%)	
Unknown primary	3 (15.0%)	3 (10.3%)	
**Liver metastasis**	8 (40.0%)	14 (48.3%)	0.8
**Brain metastasis**	7 (35.0%)	7 (24.1%)	0.5
**LDH value**	276.0 [187.5, 380.5]	329.0 [192.0, 451.0]	0.7
Missing	0	4	
**Line of therapy**			>0.9
First-line	6 (30.0%)	10 (34.5%)	
Second-line or beyond	14 (70.0%)	19 (65.5%)	
**Targeted therapy regimen**			0.2
Dabrafenib/trametinib	18 (90.0%)	24 (82.8%)	
Encorafenib/binimetinib	1 (5.0%)	5 (17.2%)	
Vemurafenib	1 (5.0%)	0 (0.0%)	

^1^ Median [Q1, Q3]; *n* (%). ^2^ Wilcoxon rank sum exact test; Fisher’s exact test; Wilcoxon rank sum test.

**Table 2:** Baseline Characteristics According to Antibiotic Exposure ±60 days.

Characteristic	ABX Within 60 Days *n* = 26 ^1^	No ABX Within 60 Days *n* = 23 ^1^	*p*-Value ^2^
**Age**	60.8 [49.2, 66.5]	56.7 [44.1, 66.4]	0.7
**Sex**			0.6
Female	8 (30.8%)	9 (39.1%)	
Male	18 (69.2%)	14 (60.9%)	
**Stage**			>0.9
2 or 3 (unresectable)	4 (15.4%)	3 (13.0%)	
4 (advanced)	22 (84.6%)	20 (87.0%)	
**BRAF status**			0.6
V600E	24 (96.0%)	21 (91.3%)	
V600K	1 (4.0%)	2 (8.7%)	
Missing	1	0	
**ECOG**			0.045
≥2	9 (36.0%)	2 (9.5%)	
0–1	16 (64.0%)	19 (90.5%)	
Missing	1	2	
**Melanoma type**			0.7
Cutaneous	21 (80.8%)	21 (91.3%)	
Mucosal	1 (3.8%)	0 (0.0%)	
Unknown primary	4 (15.4%)	2 (8.7%)	
**Liver metastasis**	10 (38.5%)	12 (52.2%)	0.4
**Brain metastasis**	9 (34.6%)	5 (21.7%)	0.4
**LDH value**	276.0 [178.0, 399.0]	329.0 [211.0, 451.0]	0.6
Missing	2	2	
**Line of therapy**			0.5
First-line	7 (26.9%)	9 (39.1%)	
Second-line or beyond	19 (73.1%)	14 (60.9%)	
**Targeted therapy regimen**			>0.9
Dabrafenib/trametinib	22 (84.6%)	20 (87.0%)	
Encorafenib/binimetinib	3 (11.5%)	3 (13.0%)	
Vemurafenib	1 (3.8%)	0 (0.0%)	

^1^ Median [Q1, Q3]; *n* (%). ^2^ Wilcoxon rank sum exact test; Fisher’s exact test; Wilcoxon rank sum test.

**Table 3:** Baseline Characteristics According to Antibiotic Exposure ±90 days.

Characteristic	ABX Within 90 Days *n* = 28 ^1^	No ABX Within 90 Days *n* = 21 ^1^	*p*-Value ^2^
**Age**	60.3 [46.2, 66.1]	58.2 [47.4, 66.4]	>0.9
**Sex**			0.8
Female	9 (32.1%)	8 (38.1%)	
Male	19 (67.9%)	13 (61.9%)	
**Stage**			>0.9
2 or 3 (unresectable)	4 (14.3%)	3 (14.3%)	
4 (advanced)	24 (85.7%)	18 (85.7%)	
**BRAF status**			0.6
V600E	26 (96.3%)	19 (90.5%)	
V600K	1 (3.7%)	2 (9.5%)	
Missing	1	0	
**ECOG**			0.092
≥2	9 (33.3%)	2 (10.5%)	
0–1	18 (66.7%)	17 (89.5%)	
Missing	1	2	
**Melanoma type**			0.8
Cutaneous	23 (82.1%)	19 (90.5%)	
Mucosal	1 (3.6%)	0 (0.0%)	
Unknown primary	4 (14.3%)	2 (9.5%)	
**Liver metastasis**	12 (42.9%)	10 (47.6%)	0.8
**Brain metastasis**	10 (35.7%)	4 (19.0%)	0.3
**LDH value**	276.0 [181.0, 385.0]	349.0 [211.0, 460.0]	0.5
Missing	2	2	
**Line of therapy**			0.2
First-line	7 (25.0%)	9 (42.9%)	
Second-line or beyond	21 (75.0%)	12 (57.1%)	
**Targeted therapy regimen**			>0.9
Dabrafenib/trametinib	24 (85.7%)	18 (85.7%)	
Encorafenib/binimetinib	3 (10.7%)	3 (14.3%)	
Vemurafenib	1 (3.6%)	0 (0.0%)	

^1^ Median [Q1, Q3]; *n* (%). ^2^ Wilcoxon rank sum exact test; Fisher’s exact test; Wilcoxon rank sum test.

## Data Availability

The data presented in this study are available upon reasonable request from the corresponding author followed by data transfer agreement.

## Supplementary Materials

The following supporting information can be downloaded at: https://www.mdpi.com/article/10.3390/curroncol32110630/s1, Table S1: Antibiotic Use and Duration by Indication Within ±30 Days of BRAFi/MEKi Initiation; Table S2: Antibiotic Use and Duration by Indication Within ±60 Days of BRAFi/MEKi Initiation; Table S3: Antibiotic Use and Duration by Indication Within ±90 Days of BRAFi/MEKi Initiation; Table S4: Frequency of Antibiotic Classes Administered Within ±30 Days of BRAFi/MEKi Initiation; Table S5: Frequency of Antibiotic Classes Administered Within ±60 Days of BRAFi/MEKi Initiation; Table S6: Frequency of Antibiotic Classes Administered Within ±90 Days of BRAFi/MEKi Initiation.

## Abbreviations

The following abbreviations are used in this manuscript:

BRAFi	BRAF V600 inhibitor
MEKi	MEK inhibitor
ATB	Antibiotic
ICI	Immune checkpoint inhibitor
ORR	Overall response rate
PFS	Progression-free survival
mPFS	Median progression-free survival
OS	Overall survival
mOS	Median overall survival
HR	Hazard ratio
CI	Confidence interval
BMT	Bone marrow transplantation
CAR-T	Chimeric antigen receptor T-cell therapy
NSCLC	Non–small cell lung cancer
RCC	Renal cell carcinoma
