# Supplementary material for: Antibiotic Exposure Does Not Impact Anti-BRAF/Anti-MEK Targeted Therapy Outcome in Patients with Advanced Melanoma
Source: Curr Oncol. 2025 Nov 10;32(11):630. doi: 10.3390/curroncol32110630 (PMC12650902; doi:10.3390/curroncol32110630)
Supplement: Supplementary file 1 [file curroncol-32-00630-s001.zip › curroncol-3934818-supplementary.pdf]

Current Oncology MDPI

Table S1. Antibiotic Use and Duration by Indication Within ±30 Days of BRAFi/MEKi Initiation.

Indication	Number of treatments	Median Duration (days, IQR)
Fever without source	19	1 [1-3]
Urinary tract infection	13	4 [2-7]
Pneumonia	8	7 [5.8-9]
Surgical prophylaxis	5	1 [1-1]
Other/Unknown indication	4	8 [4.8-10]
Clostridium difficile	4	6 [3.5-8.5]
Corticosteroid prophylaxis	3	126 [83.5-183]
Gastrointestinal infection	3	6 [4.5-9.5]
Latent tuberculosis	3	115 [81.5-118.5]
Cellulitis	2	11 [9.5-12.5]
Deep tissue infection	1	21 [21-21]

Table S2. Antibiotic Use and Duration by Indication Within ±60 Days of BRAFi/MEKi Initiation.

Indication	Number of treatments	Median Duration (days, IQR)
Fever without source	22	1 [1-3]
Urinary tract infection	14	4.5 [2.5-7]
Pneumonia	8	7 [5.8-9]
Other/Unknown indication	8	5 [1-7.8]
Surgical prophylaxis	5	1 [1-1]
Cellulitis	4	8.5 [8-10.2]
Clostridium difficile	4	6 [3.5-8.5]
Corticosteroid prophylaxis	3	126 [83.5-183]
Gastrointestinal infection	3	6 [4.5-9.5]
Latent tuberculosis	3	115 [81.5-118.5]
Deep tissue infection	1	21 [21-21]

Table S3. Antibiotic Use and Duration by Indication Within ±90 Days of BRAFi/MEKi Initiation.

Indication	Number of treatments	Median Duration (days, IQR)
Fever without source	22	1 [1-3]
Urinary tract infection	14	4.5 [2.5-7]
Pneumonia	10	8 [6.2-9.8]
Other/Unknown indication	8	5 [1-7.8]
Surgical prophylaxis	7	1 [1-1]
Cellulitis	4	8.5 [8-10.2]
Clostridium difficile	4	6 [3.5-8.5]
Corticosteroid prophylaxis	3	126 [83.5-183]
Gastrointestinal infection	3	6 [4.5-9.5]
Latent tuberculosis	3	115 [81.5-118.5]
Deep tissue infection	2	35.5 [28.2-42.8]

Table S4. Frequency of Antibiotic Classes Administered Within ±30 Days of BRAFi/MEKi Initiation.

Antibiotic Class	Number of treatments
Penicillin/beta-lactamase inhibitor	28 (35.9%)
Fluoroquinolone	12 (15.4%)
Cephalosporin	10 (12.8%)
Sulfonamide	8 (10.3%)
Glycopeptide	6 (7.7%)
Nitrofuran	3 (3.8%)
Antimycobacterial	2 (2.6%)
Antiviral	2 (2.6%)
Atovaquone	2 (2.6%)
Macrolide	2 (2.6%)
Carbapenem	1 (1.3%)
Nitroimidazole	1 (1.3%)
Penicillin	1 (1.3%)

Table S5. Frequency of Antibiotic Classes Administered Within ±60 Days of BRAFi/MEKi Initiation.

Antibiotic Class	Number of treatments
Penicillin/beta-lactamase inhibitor	32 (35.6%)
Fluoroquinolone	13 (14.4%)
Cephalosporin	11 (12.2%)
Sulfonamide	9 (10%)
Glycopeptide	6 (6.7%)
Antiviral	5 (5.6%)
Nitrofuran	3 (3.3%)
Antimycobacterial	2 (2.2%)
Atovaquone	2 (2.2%)
Carbapenem	2 (2.2%)
Macrolide	2 (2.2%)
Penicillin	2 (2.2%)
Nitroimidazole	1 (1.1%)

Table S6. Frequency of Antibiotic Classes Administered Within ±90 Days of BRAFi/MEKi Initiation.

Antibiotic Class	Number of treatments
Penicillin/beta-lactamase inhibitor	33 (33.3%)
Cephalosporin	14 (14.1%)
Fluoroquinolone	14 (14.1%)
Sulfonamide	10 (10.1%)
Glycopeptide	7 (7.1%)
Antiviral	5 (5.1%)
Carbapenem	3 (3%)
Nitrofuran	3 (3%)
Antimycobacterial	2 (2%)
Atovaquone	2 (2%)
Macrolide	2 (2%)
Nitroimidazole	2 (2%)
Penicillin	2 (2%)
